# Omics Views of Mechanisms for Cell Fate Determination in Early Mammalian Development

**DOI:** 10.1016/j.gpb.2023.03.001

**Published:** 2023-04-17

**Authors:** Lin-Fang Ju, Heng-Ji Xu, Yun-Gui Yang, Ying Yang

**Affiliations:** 1 Sino-Danish College, University of Chinese Academy of Sciences, Beijing 100049, China; 2 University of Chinese Academy of Sciences, Beijing 100049, China; 3 CAS Key Laboratory of Genomic and Precision Medicine, Collaborative Innovation Center of Genetics and Development, Beijing Institute of Genomics, Chinese Academy of Sciences and China National Center for Bioinformation, Beijing 100101, China; 4 Institute of Stem Cell and Regeneration, Chinese Academy of Sciences, Beijing 100101, China

**Keywords:** Cell fate determination, Cellular heterogeneity, Cell polarity, Single-cell omics, Mammalian preimplantation embryo

## Abstract

During mammalian preimplantation development, a totipotent zygote undergoes several cell cleavages and two rounds of **cell fate determination**, ultimately forming a mature blastocyst. Along with compaction, the establishment of apicobasal **cell polarity** breaks the symmetry of an embryo and guides subsequent cell fate choice. Although the lineage segregation of the inner cell mass (ICM) and trophectoderm (TE) is the first symbol of cell differentiation, several molecules have been shown to bias the early cell fate through their inter-cellular variations at much earlier stages, including the 2- and 4-cell stages. The underlying mechanisms of early cell fate determination have long been an important research topic. In this review, we summarize the molecular events that occur during early embryogenesis, as well as the current understanding of their regulatory roles in cell fate decisions. Moreover, as powerful tools for early embryogenesis research, **single-cell omics** techniques have been applied to both mouse and human preimplantation embryos and have contributed to the discovery of cell fate regulators. Here, we summarize their applications in the research of preimplantation embryos, and provide new insights and perspectives on cell fate regulation.

## Introduction

**Figure 1:**
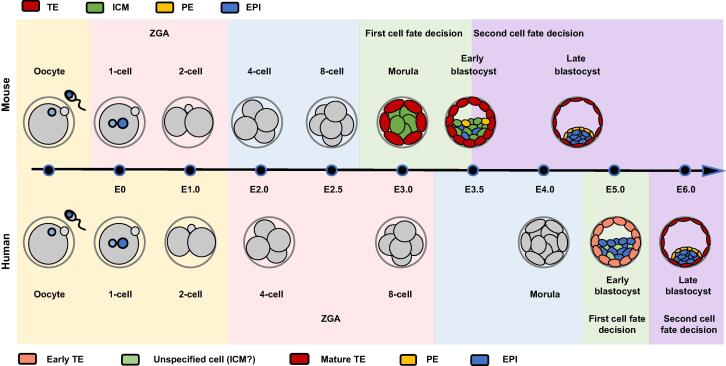
**Preimplantation embryo development in mouse and human** Schematic of the early stages of mouse (top panel) and human (bottom panel) embryonic development at different time points. The timeline in the middle indicates the embryonic days (E) from E0 to E6.0. Color legends at the top and bottom indicate the respective embryonic cells and lineages in mouse (top) and human (bottom). TE, trophectoderm; ICM, inner cell mass; PE, primitive endoderm; EPI, epiblast; ZGA, zygotic genome activation.

During mammalian preimplantation embryonic development, a fertilized egg goes through 2-cell, 4-cell, 8-cell, and morula (referring to the compacted 16- to 32-cell embryos) stages to form a hollow sphere blastocyst embryo, during which sequential events occur, including zygotic genome activation (ZGA), embryo compaction, and two rounds of cell fate determination [1] (Figure 1). Cells at the blastocyst stage comprise three cell lineages: trophectoderm (TE) for placenta, primitive endoderm (PE; a predecessor of the yolk sac), and epiblast (EPI; the progenitor for the fetus). TE cells are localized in the outer layers of the blastocyst with apical polarity, whereas cells in the center, referred to as inner cell mass (ICM) cells, consist of PE and EPI with strong cell pluripotency [2], [3], [4], [5]. The location of ICM cells delineates the “embryonic pole” of blastocyst and lies on the opposite of the “ab-embryonic pole,” demonstrating cell polarity in the blastocyst [6]. Compared with mouse embryos, lineage allocations of human preimplantation embryos are completed a little later, until cell fate decisions emerge after the cavitation of blastocysts (Figure 1). Currently, two models have been proposed to explain early human cell fate determination: one is a two-step model in which the formation of TE–ICM and TE–PE–EPI occurs in order [7], and the other is a one-step model which suggests the simultaneous occurrence of TE, EPI, and PE lineages [8]. A more recent study has reported that during human blastocyst formation, a type of unspecified cell, similar to ICM cells, emerges at the Blastocyst 2 (B2) to B3 stages, which is indicated by the EPI marker *Ifi16* and the PE marker *Gata4*. This finding provides new evidence for a two-step model [9].

Although the biological functions of transcription factors and signaling pathways at the morula and blastocyst stages have been revealed, what and how other omics events control the first two rounds of cell fate determination remain largely unknown. Currently, early cell fate determination has been reported to occur much earlier than the morula stage when morphological heterogeneity emerges. For example, the molecular basis of cell polarity at the 8-cell stage [10] and cell-to-cell differences (cellular heterogeneity) at the 2- and 4-cell stages [11], [12] have been shown to regulate cell fate decisions by functioning in downstream networks.

In this review, we summarize the molecular events that occur during early embryogenesis, as well as the current understanding of their regulatory roles in cell fate decisions. Single-cell omics techniques have been employed as powerful tools in early embryogenesis research to dissect programmed molecular events in mouse and human preimplantation embryos, and many discoveries of cell fate determination have been reported. Here, we have summarized their applications in the research field of preimplantation embryos, and new insights and future perspectives based on current omics data are also included.

## Early cell fate determination in mammalian preimplantation embryos

The first two waves of cell fate determination lead to the formation of three cell lineages. Each wave is indicated by the heterogeneous distribution of specific transcription factors and the distinctly activated states of signaling pathways.

### The first cell fate determination

Both TE and ICM cell lineages are achieved by the first cell fate determination (Figure 1), in which the representative transcription factors are activated, such as SOX2, OCT4, and NANOG in ICM and CDX2 in TE [5], [13], [14], [15], [16]. *Sox2* and *Oct4* are highly expressed at the morula and blastocyst stages and are detectable at the 2- and 4-cell stages [13], [14], [17], [18], [19], indicating that these two genes potentially regulate lineage separation at a much earlier stage. Blastocyst cavitation is not affected by lack of SOX2 but fails to survive shortly after implantation [13]. *Nanog* displays a unique expressional pattern with a predominant distribution in the ICM fate cells of morula embryos *versus* a randomly scattered distribution in the TE [14], [20]. *Cdx2* is a specific marker gene of TE in early embryogenesis and is predominantly expressed in the outer cells of morula and blastocyst than in the inner cells. Loss of *Cdx2* fails to maintain blastocoels and induces embryonic death prior to implantation [5]. Additionally, *Id2* has been identified as a specific TE cell marker by single-cell expression analysis [21].

**Figure 2:**
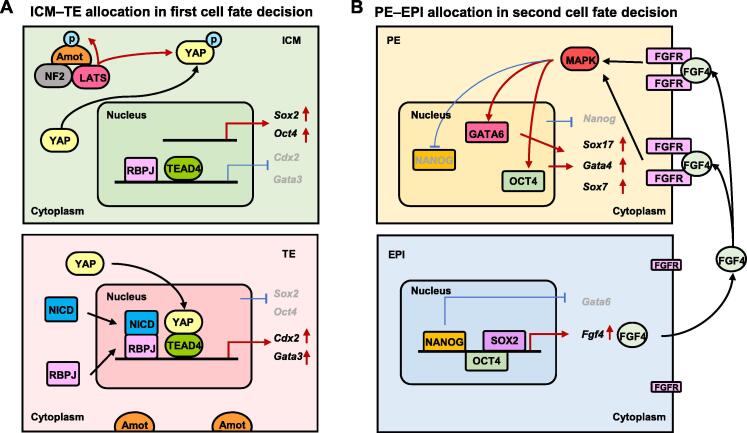
**Signaling pathways involved in ICM–TE and PE–EPI fates** **A.** Hippo and Notch signaling pathways involved in the ICM and TE cell fate allocations. In the Hippo pathway, unphosphorylated YAP enters the nucleus to interact with TEAD4, and thus activates the expression of *Cdx2* and *Gata3* in TE cells (bottom panel); phosphorylated YAP maintains in cytoplasm, resulting in the up-regulated expression of *Oct4* and *Sox2* in ICM cells (top panel). Amot regulates the activation of YAP. In the Notch pathway, NICD and RBPJ form a complex in the nucleus of TE cells, activating the expression of *Cdx2* and *Gata3*. **B.** FGF signaling pathway involved in the PE and EPI cell fate allocations. In EPI cells (bottom panel), NANOG coordinates with OCT4 and SOX2 to up-regulate *Fgf4* expression and repress *Gata6* expression. In PE cells (top panel), FGF4 secreted from EPI cells activates the FGF/MAPK signaling, and further up-regulates GATA6 and OCT4 which activate the expression of *Sox17*, *Gata4*, and *Sox7*. p, phosphorylation.

The Hippo and Notch signaling pathways drive the first cell fate determination in mammals (Figure 2A), whereas Hippo also serves as a tumor suppressor signaling pathway conserved in both mice and humans [22]. In mouse blastocysts, Hippo is activated in ICM cells but inactive in TE cells through changes in the state of its key components, TEAD4 and YAP. As an important effector of the Hippo pathway, the phosphorylation of YAP activates the Hippo pathway and favors ICM fate [23], [24]. First, NF2 (also known as Merlin) in the Hippo pathway acts upstream of LATS (LATS1/2) kinases; second, another effector of Hippo, junction-associated scaffolding angiomotin (Amot), is phosphorylated by activated LATS; third, the activated NF2, LATS, and Amot form a regulatory complex, further phosphorylating YAP for its retention in the cytoplasm, which results in the high expression of *Sox2* and *Oct4* and facilitates ICM lineage specificity [25], [26]. In TE cells, Hippo is inactive; thus, YAP and Amot are in an unphosphorylated state. Unphosphorylated Amot localizes to the apical domain without functioning in YAP [27]. Unphosphorylated YAP translocates from the cytoplasm to the nucleus and interacts with TEAD4 to up-regulate the expression of *Cdx2* and *Gata3*, promoting cell differentiation into the TE lineage [28], [29]. The Notch signaling pathway controls the first cell fate determination by cooperating with YAP and TEAD4 [30] (Figure 2A). In TE cells, both the YAP–TEAD4 complex of Hippo and the NICD–RBPJ complex of Notch enter the nucleus and up-regulate the expression of TE-specific genes, such as *Cdx2*, to promote TE fate [31], [32].

### The second cell fate determination

After the first cell fate determination, ICM cells further differentiated into EPI and PE cells in the second cell fate determination. *Nanog* and *Gata6*, serving as the specific markers for EPI and PE, respectively, are required for this process [33], [34]. Although both begin to be expressed at the 8-cell stage [35], the balance of expression is not tipped until ICM formation. In blastocyst embryos, *Nanog*^+^/*Gata6*^−^ results in EPI fate, whereas *Gata6*^+^/*Nanog*^−^ leads to PE fate, referring to a typical “salt-and-pepper” distribution model. Additionally, other transcription factor-coding genes, such as the downstream genes of *Gata6* including *Sox17*, *Gata4*, and *Sox7*, were also found to be intimately correlated with the maturation of PE [36].

The FGF signaling pathway regulates the second cell fate determination in mammalian embryos [1], [27], [37] (Figure 2B). When the second cell fate determination is initiated, ICM cells express *Nanog* and *Gata6* in a salt-and-pepper manner, in which relatively high expression levels of *Gata6* and *Nanog* separately prompt the expression of *Fgfr* [a gene encoding fibroblast growth factor receptor (FGFR)] and *Fgf4* [a gene encoding fibroblast growth factor 4 (FGF4)] [38]. NANOG collaborates with OCT4 and SOX2 to up-regulate the expression of *Fgf4* and represses the expression of *Gata6*, leading to the secretion of FGF4 and bias of EPI [1]. FGF4 specifically interacts with FGFR and activates the FGF/MAPK signaling cascade in PE cells, resulting in up-regulation of *Gata6* and inhibition of *Nanog* [1], [33]. Subsequently, activated FGF signaling and GATA6 cooperatively up-regulate the downstream genes of GATA6 and OCT4, including *Sox17*, *Gata4*, and *Sox7* [27]. After separation of PE and EPI, specified PE progenitors express apical polarity proteins and form an epithelial layer to prevent further cell mixing [35], [39].

Additionally, based on single-cell RNA sequencing (scRNA-seq) analysis in 16-cell embryos, BMP signaling was shown to regulate the correct development of TE and PE fates. In blastocysts, the BMP ligands, *Bmp4* and *Bmp7*, are specifically expressed in ICM cells, whereas the BMP receptor *Bmpr2* is predominantly distributed in TE cells. Repressing BMP activity by blocking *Smad4* or *Bmpr2* results in a significant reduction in the cell numbers of TE and PE, but not embryonic EPI. Depletion of *Bmp4* and *Bmp7* significantly decreases the cell numbers of TE and PE, but not EPI [40]. This discovery indicates that BMP signaling is important for the development of extra-embryonic TE and PE lineages, expanding the knowledge of key signaling pathways in early cell fate decisions.

Most of the transcription factors interact or influence each other and coordinate with the signaling pathways of Hippo, Notch, and FGF, to form a functional network modulating the early cell fate decisions.

## Establishment of apicobasal cell polarity: symmetry-breaking

When the compaction process is initiated, the blastomeres of the embryo break the symmetry of cell morphology (symmetry-breaking). And owing to the increased cell–cell adhesion and altered cortex tension, both obviously different cell morphology and cell polarization emerge at the 8-cell stage. Molecule-based cell polarity interacts with signaling pathways to regulate cell lineage allocation, including spindle assembly and glycolysis.

### Molecular basis of cell polarity establishment

Cell polarity is axially established from the center to the surface, dividing blastomeres into polar cells with non-polar cells residing in the outer and central areas [7], [41], [42]. After compaction, most cells of mid- or late 8-cell embryos give rise to a polar cell and a non-polar cell at the 16-cell stage or two polar cells in which one is internalized into the inner cell by the apical constriction driven by cortical tension [43]. Inner cells remain unpolarized and express pluripotency-associated factors, generating ICM cells in blastocysts [4]. The outer cells polarize their apical cortex and establish a cortical F-actin ring consisting of apical polarity proteins, such as PAR6B, ezrin, and keratin, which further triggers the differential regulation of the Hippo effector YAP and the transcription factor-coding gene *Cdx2*, benefiting TE cell fate. Thus, this internalization event interacts with the Hippo signaling pathway to regulate cell fate [25], [44], [45], [46].

Based on scRNA-seq and RNA interference (RNAi) experiments, transcription factor-coding genes *Tfap2c* and *Tead4* were found to be essential for embryo polarization at the 8- to 16-cell stages. Mechanically, TFAP2C and TEAD4 recruit ezrin, promoting the polarization growth of apical protein clusters, which eventually leads to apical protein centralization and subsequent regulation of apical formation and lineage allocation through positive feedback with RhoA [10].

The regulation of cell polarity on cell fate determination seems to imply that components in polarity cells also participate in cell fate control by asymmetric inheritance during cell division, even though lineage specification driven by asymmetrically inherited fate determinants is more widely accepted in non-mammalian embryos than in mammalian embryos [47]. Keratin participates in the regulation of cell polarity, Hippo signaling pathway, and mechanisms in epithelial tissues [48]. In mouse early 8-cell embryos, keratin-containing cells are asymmetrically inherited during cell division, leading to the formation of inner daughter cells with fewer keratins and outer daughter cells with more keratins. As a TE cell marker [49], more keratins anchor to the apical domain and promote apical polarization and YAP-dependent expression of *Cdx2*, prompting cells with more keratins to differentiate into TE cells [50].

### Downstream regulatory network of cell polarity establishment

Spindle assembly plays a role in regulating cell fate through cell polarity and keratin. Because of the lack of a microtubule-organizing center (MTOC) to generate microtubule asters in mammalian preimplantation embryos [51], [52], whether spindle organization regulates early mammalian lineage segregation remains elusive. A recent study reported that cellular heterogeneities of cell polarity in early mouse 8-cell embryos activate an asymmetric spindle assembly, which forms a single microtubule aster (monoastral spindle) from the apically localized non-centrosomic MTOC in an unusual manner. The entirely assembled spindle attaches to the residual apparatus and activates a spatially asymmetrical pattern of division, separating cells inside and outside locations. After mitosis, pulling toward the cortex in the apical regions of the monoastral spindle triggers a burst of F-actin and myosin II, delivering microtubules to the cortex in which the F-actin ring is established and apical polarity proteins are enriched, which retain TE-specified cells in the peripheral position. In contrast, basal regions with an anastral spindle do not clear F-actin or form a ring, internalizing into the central position. Moreover, cellular heterogeneity in keratin assembly differentially triggers the formation of monoastral spindles by stabilizing the cell cortex in some blastomeres of 8-cell embryos. The outer cells with rings and monoastral spindles displayed a higher level of YAP protein, and disruption of F-actin reduced *Cdx2* expression, fully proving that the monoastral spindle regulates TE/ICM lineage segregation [53]. These results suggest that asymmetric spindle organization modulates the choice of ICM–TE fate.

Glucose and glycolysis also modulate TE and ICM separation at the polarized morula stage. In TE fate cells of morula embryos, the hexosamine biosynthetic pathway (HBP) responsible for glucose metabolism allows YAP1 to localize to the nucleus. The pentose phosphate pathway (PPP), which participates in glucose-dependent nucleotide synthesis, combines with sphingolipid (S1P) signaling to activate the mTOR pathway and allow the translation of *Tfap2c*. Ultimately, YAP1, TEAD4, and TFAP2C form a heterotrimer that functions as a transcription factor to activate TE-specific markers such as *Cdx2*, implicating that glucose signaling specifically controls TE cell fate rather than ICM fate specification in early embryos [54].

## Cellular heterogeneity: before symmetry-breaking

As mentioned in the section above, the expression patterns of transcription factor-coding genes such as *Sox2*, *Nanog*, *Cdx2*, and *Gata6* display significant heterogeneity with distinct cell lineages of ICM–TE or PE–EPI–TE during early cell fate decision processes [55]. Cellular heterogeneities at the earlier developmental stages (including the 2- and 4-cell stages) also participate in subsequent cell fate decisions by their functions on those specific transcription factors [11], [12], [56]. The regulatory relationship between cell fate decisions and cellular heterogeneities, including heterogeneous histone H3 methylation at arginine 26 (H3R26me) and heterogeneous transcription factor kinetics, is discussed in the following section.

### Heterogeneous H3R26me

The epigenetic reprogramming process is essential for sustaining pluripotency in early mouse embryogenesis [57], [58]. Many cell fate regulators reported in earlier stages are closely related to a type of histone methylation: H3R26me.

H3R26me and its methyltransferase CARM1 display significant cell-to-cell variations in mouse 4-cell embryos with equatorial and meridional division of the zygote [56]. Overexpression of *Carm1* increases the abundance of H3R26me and the expression of *Nanog*, *Sox2*, and *Sox21*, indicating that CARM1–H3R26me instructs cell fate decisions by promoting the expression of transcription factors [56], [59]. Interestingly, CARM1 localizes to a nuclear paraspeckle consisting of p54nrb, PSPC1, PSF, and *LncNEAT1*. The number of paraspeckles is differentially accumulated in nuclei of 2- to 4-cell embryos as well [60]. Depletion of p54nrb or *LncNEAT1* leads to failed blastocyst cavitation and elevated expression of *Cdx2*, promoting cell differentiation into the TE fate [56], [60]. All aforementioned studies reveal a CARM1–H3R26me-mediated mechanism of epigenetic manipulation on cell fate determination: in mouse 4-cell embryos, cells with high levels of CARM1 and H3R36me favor ICM fate, and cells with low levels of CARM1 and H3R26me direct developmental fate to TE. In addition, CARM1 also regulates cell fate by affecting cell polarity and keratin allocation [61].

Similar to *Carm1*, heterogenous *Prdm14* controls cell lineage in 4-cell embryos. PRDM14, a PR-domain and zinc finger protein, is only distributed in early embryonic tissues and during reprogramming events [62], [63], [64]. Using single-cell quantitative combinatorial expression profiling of chromatin modifiers, *Prdm14* was observed to have heterogeneous expression at the mouse 4-cell stage. Mechanistically, PRDM14 interacts with CARM1 to drive progenies toward pluripotent cells by increasing H3R26me in mouse 4-cell embryos [65].

The aforementioned cellular heterogeneities in CARM1 and CARM1-paraspeckles occur at the 4-cell stage; however, the key questions are what induces these differences and whether there is cellular heterogeneity associated with early cell fate decision before the 4-cell stage. A long non-coding RNA, *LincGET*, provides an answer [11]. *LincGET*, serving at the upstream of the CARM1–H3R26me axis, presents asymmetric expression starting at the late 2-cell stage and ending at the 8-cell stage in mice. Depletion of *LincGET* leads to developmental arrest at the 2-cell stage in early mouse embryos. Overexpression of *LincGET* or *Carm1* increases chromatin accessibility and expression of ICM-specific genes. Mechanistically, *LincGET* interacts and forms a complex with CARM1 to increase the level of H3R26me, which then activates the chromatin accessibility of ICM-specific genes and biases blastomeres with higher *LincGET* levels toward ICM fate. Conversely, blastomeres with lower *LincGET* levels preferentially differentiate into TE cells [11].

Thus, *LincGET*–CARM1 (in paraspeckle)–H3R26me forms a robust regulatory axis to modulate the subsequent cell fate decision at the 2- to 4-cell stages.

### Heterogeneous kinetics of transcription factors

Cellular differences in the dynamic kinetics of transcription factors lead to their differential accessibility to DNA targets, which in turn are controlled by differential epigenetic regulation and participate in early cell fate determination.

Transcription factor-coding genes, *Oct4* and *Sox2*, are highly expressed in the ICM lineage at the morula and blastocyst stages and also exhibit detectable expression in earlier stages, implying that these two transcription factors are involved in early cell fate regulation at an earlier time in a way that is different from their expression. The biological activity of transcription factors *in vivo* strongly depends on their kinetic behaviors, which effectively modulates gene expression and cell fate [66], [67]. Using a fluorescence decay after photoactivation (FDAP) assay, two distinct kinetic behaviors of OCT4 resulting from differences in OCT4 accessibility to its DNA targets were observed in mouse 4-cell embryos. These results further point to two distinct cell lineages by lineage tracing: cells with slower OCT4 kinetics are more likely to give rise to the pluripotent cell lineage that contributes to ICM fate. Conversely, cells with faster OCT4 kinetics mostly differentiate into TE fate [68]. Similar to OCT4, the binding kinetics of SOX2 to DNA is also found to participate in lineage specificity and cell fate choice by an FDAP assay [17]. Long-lived binding of SOX2 corresponds to higher CARM1 and H3R26me levels in the same blastomeres of 4-cell embryos. *Carm1* deficiency significantly decreases the long-lived bound fraction of SOX2 and the expression of *Sox2*, *Nanog*, *Oct4*, and *Sox2* targets. Thus, blastomeres in 4-cell embryos with long-lived SOX2 binding mode preferentially bias their progenies to ICM cells [17].

**Figure 3:**
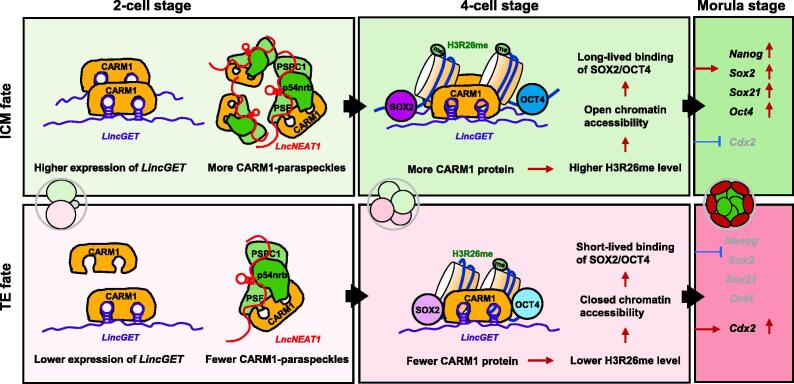
**Cellular heterogeneities of cell fate regulators control differential fate decision** A schematic of the heterogeneous levels of fate regulators at the 2- and 4-cell stages contribute to the heterogeneous expression of lineage transcription factors at the morula stage. Left: asymmetric *LincGET* expression and differential CARM1-containing paraspeckles are present in blastomeres of 2-cell embryos. Middle: heterogeneous CARM1, H3R26me, and OCT4/SOX2 kinetics are present in the blastomeres of 4-cell embryos. Right: regulations by transcription factors are presented at the blastomeres of morula embryos. H3R26me, histone H3 methylation at arginine 26.

Taken together, we describe a mechanistic model of cellular heterogeneities to explain the cell fate decision during the developmental process from 2-cell to morula stages (Figure 3). (1) The expression of *LincGET* and the number of CARM1-containing nuclear paraspeckles are asymmetrically distributed in 2- and 4-cell embryos. (2) *LincGET* coupling with heterogeneous CARM1 and CARM1-containing paraspeckles differentially regulates H3R26me in 4-cell embryos, leading to distinct chromatin accessibility at the 4-cell stage. (3) The opened chromatin benefits the long-lived binding of OCT4 and SOX2 to DNA in 4-cell embryos, resulting in the subsequent increased transcription of ICM-related target genes, including *Nanog*, *Oct4*, *Sox2*, and *Sox21*, biasing cells to ICM fate. Conversely, lower expression of *LincGET*, together with fewer CARM1 and CARM1-containing paraspeckles, decreases H3R26me levels and further results in the short-lived DNA binding of OCT4/SOX2, leading to the low expression levels of their targets and activation of TE-specific markers, which predominantly direct cell differentiation into TE cells.

## Application of single-cell omics to early cell fate determination

Many of the aforementioned molecular events involved in early cell fate determination were discovered by single-cell transcriptomics or single-cell expression profiles, such as the outer cell-specific marker *Id2* at the morula stage, differentiated BMP signaling at the morula and blastocyst stages, regulation of *Tfap2c* and *Tead4* on cell polarity at the 8- to 16-cell stages, heterogeneous *Prdm14* at the 4-cell stage, and asymmetric *LincGET* at the 2-cell stage [10], [11], [40], [65]. Thus, single-cell omics is a feasible and robust tool to study cell fate determination during early mammalian embryogenesis.

### Single-cell transcriptome

Benefiting from the application of next-generation sequencing platform-based scRNA-seq in mouse preimplantation embryos, dozens of protein-coding genes showed bimodal expression patterns in mouse 2- and 4-cell embryos, and their co-expression exhibited high association with TE and ICM cell fate commitment [69]. Similarly, analyses based on the single-blastomere transcriptome data of mouse and human preimplantation embryos also revealed that the earliest symmetry-breaking and initial cell-to-cell biases emerge at the first embryonic cleavage division with a binomial distribution pattern [70]. Cell-to-cell transcriptional variations were further elevated with the subsequent development of embryos. During the preimplantation process, some genes with the function of lineage specifiers showed ever-increasing asymmetry between blastomeres, whereas others showed a minimized inter-cell difference, suggesting that transcriptional regulation of symmetry-breaking and early cell fate determination contains complicated positive and negative feedbacks by the differential allocation of two types of genes in early mammalian embryos [70]. In mouse 4-cell embryos, scRNA-seq revealed that the target genes of OCT4 and SOX2 displayed highly heterogeneous expression patterns, in which *Sox21* is the one showing the most heterogeneous expression profile [71], [72]. Depletion of *Sox21* contributes to greater TE fate than ICM fate by up-regulating the expression and protein levels of *Cdx2*, indicating that *Sox21* benefits ICM cell fate. Intriguingly, the expression of *Sox21* and other ICM-related genes, such as *Nanog* and *Esrrb*, is influenced by CARM1. The increased or inhibited CARM1 corresponds to up-regulation or down-regulation of *Sox21*, indicating that CARM1-containing epigenetic regulation acts upstream of these genes in cell fate determination [59].

In short, scRNA-seq analysis uncovered inter-cell transcriptional variabilities appearing at the 2- to 4-cell stages in early mouse embryos [11], [59], [65], [69], [70]. These cellular heterogeneities affect the first cell fate decision in mouse preimplantation development by inducing other biological processes such as cell division, gene expression, and epigenetic modification.

### Other single-cell omics techniques

Except for single-cell transcriptomics, omics at different layers, including DNA methylation, chromatin architecture, RNA methylation, and RNA translation, is yet to be explored in their regulatory roles in cellular heterogeneity and early cell fate determination during early mammalian embryogenesis, despite the fact that their dynamic landscapes and regulations in epigenetic remodeling have already described. The single-cell omics and multi-omics techniques available for research on mammalian preimplantation embryos are summarized in Table 1. Considering the similarities in both size and volume between mature mammalian oocytes and single cells of early preimplantation embryos, single-cell sequencing techniques applied to oocytes are included in this section as well.

**Table 1:** **Single-cell sequencing techniques applied to mouse and human preimplantation embryos**

**Type of omics**	**Single-cell sequencing technique**	**Applied to preimplantation embryos**	**Refs.**
Transcriptome	scRNA-seq	Mouse/human oocytes; mouse/human zygotes, 2-cell, 4-cell, 8-cell, morula, and blastocyst embryos	[8], [9], [40], [59], [69], [99], [100], [101], [102], [103], [104], [105], [106], [107], [108], [109], [110], [111], [112]
	SCAN-seq	Mouse oocytes; mouse zygotes, 2-cell, 4-cell, 8-cell, morula, and blastocyst embryos	[113]
DNA methylatome (5mC)	scRRBS	Mouse oocytes; mouse zygotes	[73]
	scBS-seq	Mouse oocytes	[74]
	scPBAT-seq	Human oocytes; human zygotes, 2-cell, 4-cell, 8-cell, morula, and blastocyst embryos	[75]
	scMspJI-seq	Mouse 2-cell, 4-cell, 8-cell, morula, and blastocyst embryos	[76]
DNA methylatome (5fC)	scCLEVER-seq	Mouse oocytes; mouse 2-cell, 4-cell, and blastocyst embryos; human zygotes, 2-cell, 4-cell, 8-cell, morula, and blastocyst embryos	[79]
			[80]
Chromatin architecture	snHi-C	Mouse oocytes; mouse zygotes	[83]
	scHi-C	Mouse zygotes, 2-cell, 4-cell, 8-cell, and blastocyst embryos	[84]
RNA methylatome (m^6^A)	scm^6^A-seq	Mouse oocytes; mouse 2-cell and 4-cell embryos	[85]
Transcriptome and DNA methylome	scM&T-seq	Human blastocyst embryos	[93]
DNA methylome and chromatin accessibility	scCOOL-seq	Mouse oocytes; mouse/human zygotes, 2-cell, 4-cell, 8-cell, morula, and blastocyst embryos	[90]
			[91]
	iscCOOL-seq	Mouse oocytes	[92]
Transcriptome, DNA methylome, and chromatin accessibility	scNOMeRe-seq	Mouse zygotes, 2-cell, 4-cell, 8-cell, morula, and blastocyst embryos	[94]
	scChaRM-seq	Human oocytes	[95]
Transcriptome and translatome	T&T-seq	Mouse/human oocytes	[88]

*Note*: 5mC, DNA 5-methylcytosine; 5fC, DNA 5-formylcytosine; m^6^A, RNA *N*^6^-methyladenosine; scRNA-seq, single-cell RNA sequencing; SCAN-seq, single-cell amplification and sequencing of full-length RNAs by Nanopore platform; scRRBS, single-cell reduced representation bisulfite sequencing; scBS-seq, single-cell bisulfite sequencing; scPBAT-seq, single-cell post-bisulfite adaptor tagging DNA methylome sequencing; scMspJI-seq, single-cell MspJI-based strand-specific 5mC sequencing method; scCLEVER-seq, single-cell chemical-labeling-enabled C-to-T conversion sequencing; snHi-C, single-nucleus high-throughput/resolution chromosome conformation capture; scHi-C, single-cell high-throughput/resolution chromosome conformation capture; scm^6^A-seq, single-cell m^6^A sequencing; scM&T-seq, single-cell genome-wide methylome and transcriptome sequencing; scCOOL-seq, single-cell chromatin overall omic-scale landscape sequencing; iscCOOL-seq, improved scCOOL-seq; scNOMeRe-seq, single-cell nucleosome occupancy, DNA methylation, and RNA expression sequencing; scChaRM-seq, single-cell chromatin accessibility, RNA barcoding, and DNA methylation sequencing; T&T-seq, transcriptome and translatome sequencing.

DNA methylation, usually referred to as 5-methylcytosine (5mC) methylation, plays a vital role in early mammalian embryogenesis [73], [74], [75], [76]. The acquisition of a whole-scale DNA methylome is frequently based on bisulfite treatment followed by sequencing (BS-seq) using a large amount of input material [77]. Advances in some techniques, such as single-cell reduced representation bisulfite sequencing (scRRBS), single-cell post-bisulfite adaptor tagging DNA methylome sequencing (scPBAT-seq), single-cell BS-seq (scBS-seq), and single-cell MspJI-based strand-specific 5mC sequencing method (scMspJI-seq), have overcome the inherent difficulty of input cells, through which single-cell dynamic DNA methylation landscapes in early mouse and human embryogenesis have been successfully obtained [73], [74], [75], [76]. In mouse 4-cell embryos, genetic lineage can be traced by DNA 5mC [75]. As an oxidized derivative of 5mC, 5-formylcytosine (5fC) regulates gene expression in maternal and paternal genomes in early mammalian embryos [78]. Single-cell chemical-labeling-enabled C-to-T conversion sequencing (scCLEVER-seq) was developed to reveal the single-cell landscape of DNA 5fC and provides important resources for functional exploration of epigenetic reprogramming in single cells and studies related to ten-eleven translocation protein family (TET)-dependent active demethylation during early mammalian embryonic development [79], [80].

High-throughput/resolution chromatin conformation capture (Hi-C) was developed to probe the three-dimensional architecture of the whole genome in the nucleus by identifying long-range interaction loci with deep sequencing [81]. Single-nucleus or single-cell Hi-C (snHi-C/scHi-C) reveals high-order chromatin and chromatin conformation profiles in mouse oocytes, zygotes, and blastomeres of preimplantation embryos [82], [83], [84], which have provided us with a powerful tool for deciphering cell-to-cell variability of chromatin architecture and cell fate determination.

RNA *N*^6^-methyladenosine (m^6^A), the most abundant RNA methylation, plays a critical role in cellular regulation and function. Benefiting from the single-cell m^6^A sequencing (scm^6^A-seq) technique, single-cell landscapes of m^6^A methylome and transcriptome have been profiled simultaneously in mouse oocytes and single blastomeres of early mouse embryos. Interestingly, m^6^A-dependent asymmetries emerge in the blastomeres of 2-cell embryos, possibly indicating the potential significance of m^6^A in early cell fate determination [85].

Although single-cell transcriptomics methods were developed over a decade ago, translatomics at single-cell resolution was not achieved until 2021, when Vanlnsberghe et al. and Brannan et al. invented single-cell ribosome sequencing (scRibo-seq) and surveying targets by APOBEC-mediated profiling of ribosomes (Ribo-STAMP) separately [86], [87]. These two methods are high-throughput and are available for cell lines rather than for preimplantation embryonic cells. More recently, dual-omics methodology of single oocyte, transcriptome and translatome sequencing (T&T-seq), successfully characterized the single-cell profiles of the transcriptome and translatome simultaneously during mouse and human oocyte maturation [88]. These single-cell techniques for the translatome offer robust tools for future investigation of both cell-to-cell translational differences and the mechanisms of translational regulation during early mammalian embryogenesis.

Multi-omics profiling, which integrates the genome, transcriptome, epigenome, and proteome, provides a powerful approach to simultaneously capture multi-layer profiles. The integrated multi-omics database DevOmics also provides a convenient tool for investigators to study the molecular regulators and relative mechanisms in early mouse and human embryos [89]. Single-cell chromatin overall omic-scale landscape sequencing (scCOOL-seq) can simultaneously analyze chromatin state, nucleosome positioning, DNA methylation, copy number variation, and ploidy in the same individual mammalian cell. Using scCOOL-seq, the dynamics and heterogeneity of DNA methylation have been described in the preimplantation embryos of mice and humans [90], [91]. Improved scCOOL-seq (iscCOOL-seq) also dissects complex epigenetic alterations during mouse oocyte growth [92]. The combined approach of the whole-scale transcriptome with DNA methylome, single-cell genome-wide methylome and transcriptome sequencing (scM&T-seq), enables the identification of susceptibility to glucocorticoids in human blastocyst embryos [93]. Single-cell nucleosome occupancy, DNA methylation, and RNA expression sequencing (scNOMeRe-seq) [94] and single-cell chromatin accessibility, RNA barcoding, and DNA methylation sequencing (scChaRM-seq) [95] are tripartite-omics techniques, which are capable of simultaneously acquiring the profiles of the DNA methylome, transcriptome, and chromatin accessibility in single blastomeres in mouse preimplantation embryos (where genetic lineages were remodeled by DNA methylation in 4- and 8-cell embryos) and in single human oocytes, respectively. All these techniques provide important resources for comprehensively understanding the functional regulatory landscape in early mammalian embryos.

Although single-cell omics and single-cell multi-omics techniques, including DNA methylome, chromatin architecture, chromatin accessibility, RNA methylome, and transcriptome, have been utilized for analyzing dynamic landscapes of early mammalian embryos, few studies so far have focused on early cell-to-cell heterogeneity and cell fate determination, except transcriptome, which are worthy of further investigation.

## Concluding remarks and perspectives

Spatial and temporal accuracy in early embryogenesis is crucial for subsequent fetal development; thus, early cell fate decisions are a matter of widespread interest. In mice, the first two rounds of cell fate are determined by transcription factors and signaling pathways at the morula and blastocyst stages, which are also affected by the establishment of cell polarity at 8-cell stage. Cell fate regulation at the 2- to 4-cell stages is characterized by cellular heterogeneities in RNA transcription, histone modification, and transcription factor kinetics. Although numerous studies have extended our understanding of cell fate determination during early embryonic development, many questions remain unanswered. For example, is there other important regulatory layers, such as RNA translation, asymmetrically distributed at the 2- to 4-cell stages or during an earlier stage and associated with early cell fate segregation? Do other types of histone modifications, such as histone acetylation, mediate distinct regulatory axes to determine cell fate? What is the origin of cell-to-cell heterogeneity, and whether this type of heterogeneity occurs randomly or procedurally? As the development of early embryos is punctual and regular, what mechanisms drive the switching between different lineage patterns and adjust the mismatch between the position and fate of a certain cell? In view of the powerful cell totipotency and embryonic plasticity in mouse 2- and 4-cell embryos [96], [97], [98], whether the molecular asymmetry can be inherited or reconstructed in new 2- and 4-cell embryos developed from “half”- and “one-quarter”-cleaving embryos is an exciting scientific question waiting to be explored. The answers to these questions will provide a clear view of the detailed mechanisms for early cell fate regulation.

Currently, single-cell omics/multi-omics techniques enable the accessibility of genetic and epigenetic profiles, which facilitates a comprehensive understanding of the development of early embryos. Except for single-cell transcriptomes, more single-cell technologies at different layers, such as RNA modifications, RNA translation, and proteome, are waiting to be innovated, integrated, and applied to explore the cell fate decision of early embryos. Advanced techniques for capturing subcellular structures may pave the way for parsing intra-cell heterogeneity caused by asymmetric molecular distribution.

Additionally, despite the fact that mouse and human preimplantation embryonic development are relatively conserved in many mechanisms and remarkably similar in morphogenesis, the understanding of early human embryogenesis is still rare. Most mechanisms and molecular events involved in early cell fate determination have been studied in mouse models. However, human lineage specification and blastocyst formation using contradictory models remain elusive. Moreover, compared with mouse embryos, the ZGA process occurs later, and the duration of cell totipotency spans longer in human embryos. Considering the rarity and precision of human samples, more mature, stable, and applicable single-cell manipulation and sequencing techniques are required to solve these problems in the future.

## Competing interests

The authors have declared no competing interests.

## CRediT authorship contribution statement

**Lin-Fang Ju:** Writing – original draft, Writing – review & editing, Visualization. **Heng-Ji Xu:** Writing – original draft, Writing – review & editing. **Yun-Gui Yang:** Conceptualization, Supervision. **Ying Yang:** Writing – review & editing, Supervision. All authors have read and approved the final manuscript.

## References

[1] Zhu M., Zernicka-Goetz M. (2020). Principles of self-organization of the mammalian embryo. Cell.

[2] Sutherland A.E., Calarco-Gillam P.G. (1983). Analysis of compaction in the preimplantation mouse embryo. Dev Biol.

[3] Houliston E., Maro B. (1989). Posttranslational modification of distinct microtubule subpopulations during cell polarization and differentiation in the mouse preimplantation embryo. J Cell Biol.

[4] Johnson M.H., Ziomek C.A. (1981). The foundation of two distinct cell lineages within the mouse morula. Cell.

[5] Strumpf D., Mao C.A., Yamanaka Y., Ralston A., Chawengsaksophak K., Beck F. (2005). *Cdx2* is required for correct cell fate specification and differentiation of trophectoderm in the mouse blastocyst. Development.

[6] Bischoff M., Parfitt D.E., Zernicka-Goetz M. (2008). Formation of the embryonic-abembryonic axis of the mouse blastocyst: relationships between orientation of early cleavage divisions and pattern of symmetric/asymmetric divisions. Development.

[7] Ziomek C.A., Johnson M.H. (1980). Cell surface interaction induces polarization of mouse 8-cell blastomeres at compaction. Cell.

[8] Petropoulos S., Edsgard D., Reinius B., Deng Q., Panula S.P., Codeluppi S. (2016). Single-cell RNA-seq reveals lineage and X chromosome dynamics in human preimplantation embryos. Cell.

[9] Meistermann D., Bruneau A., Loubersac S., Reignier A., Firmin J., Francois-Campion V. (2021). Integrated pseudotime analysis of human pre-implantation embryo single-cell transcriptomes reveals the dynamics of lineage specification. Cell Stem Cell.

[10] Zhu M., Cornwall-Scoones J., Wang P., Handford C.E., Na J., Thomson M. (2020). Developmental clock and mechanism of *de novo* polarization of the mouse embryo. Science.

[11] Wang J., Wang L., Feng G., Wang Y., Li Y., Li X. (2018). Asymmetric expression of *LincGET* biases cell fate in two-cell mouse embryos. Cell.

[12] Hupalowska A., Jedrusik A., Zhu M., Bedford M.T., Glover D.M., Zernicka-Goetz M. (2018). CARM1 and paraspeckles regulate pre-implantation mouse embryo development. Cell.

[13] Avilion A.A., Nicolis S.K., Pevny L.H., Perez L., Vivian N., Lovell-Badge R. (2003). Multipotent cell lineages in early mouse development depend on SOX2 function. Genes Dev.

[14] Nichols J., Zevnik B., Anastassiadis K., Niwa H., Klewe-Nebenius D., Chambers I. (1998). Formation of pluripotent stem cells in the mammalian embryo depends on the POU transcription factor Oct4. Cell.

[15] Chambers I., Colby D., Robertson M., Nichols J., Lee S., Tweedie S. (2003). Functional expression cloning of Nanog, a pluripotency sustaining factor in embryonic stem cells. Cell.

[16] Mitsui K., Tokuzawa Y., Itoh H., Segawa K., Murakami M., Takahashi K. (2003). The homeoprotein Nanog is required for maintenance of pluripotency in mouse epiblast and ES cells. Cell.

[17] White M.D., Angiolini J.F., Alvarez Y.D., Kaur G., Zhao Z.W., Mocskos E. (2016). Long-lived binding of Sox2 to DNA predicts cell fate in the four-cell mouse embryo. Cell.

[18] White M.D., Bissiere S., Alvarez Y.D., Plachta N. (2016). Mouse embryo compaction. Curr Top Dev Biol.

[19] Wicklow E., Blij S., Frum T., Hirate Y., Lang R.A., Sasaki H. (2014). HIPPO pathway members restrict SOX2 to the inner cell mass where it promotes ICM fates in the mouse blastocyst. PLoS Genet.

[20] Bissiere S., Gasnier M., Alvarez Y.D., Plachta N. (2018). Cell fate decisions during preimplantation mammalian development. Curr Top Dev Biol.

[21] Guo G., Huss M., Tong G., Wang C., Sun L., Clarke N.D. (2010). Resolution of cell fate decisions revealed by single-cell gene expression analysis from zygote to blastocyst. Dev Cell.

[22] Yu F.X., Guan K.L. (2013). The Hippo pathway: regulators and regulations. Genes Dev.

[23] Ma S., Meng Z., Chen R., Guan K.L. (2019). The Hippo pathway: biology and pathophysiology. Annu Rev Biochem.

[24] Frum T., Ralston A. (2015). Cell signaling and transcription factors regulating cell fate during formation of the mouse blastocyst. Trends Genet.

[25] Cockburn K., Biechele S., Garner J., Rossant J. (2013). The Hippo pathway member Nf2 is required for inner cell mass specification. Curr Biol.

[26] Lorthongpanich C., Messerschmidt D.M., Chan S.W., Hong W., Knowles B.B., Solter D. (2013). Temporal reduction of LATS kinases in the early preimplantation embryo prevents ICM lineage differentiation. Genes Dev.

[27] Lanner F. (2014). Lineage specification in the early mouse embryo. Exp Cell Res.

[28] Yagi R., Kohn M.J., Karavanova I., Kaneko K.J., Vullhorst D., DePamphilis M.L. (2007). Transcription factor TEAD4 specifies the trophectoderm lineage at the beginning of mammalian development. Development.

[29] Ralston A., Cox B.J., Nishioka N., Sasaki H., Chea E., Rugg-Gunn P. (2010). Gata3 regulates trophoblast development downstream of Tead4 and in parallel to Cdx2. Development.

[30] Rayon T., Menchero S., Nieto A., Xenopoulos P., Crespo M., Cockburn K. (2014). Notch and Hippo converge on Cdx2 to specify the trophectoderm lineage in the mouse blastocyst. Dev Cell.

[31] Wang H., Zang C., Liu X.S., Aster J.C. (2015). The role of Notch receptors in transcriptional regulation. J Cell Physiol.

[32] Yao C., Zhang W., Shuai L. (2019). The first cell fate decision in pre-implantation mouse embryos. Cell Regen.

[33] Chazaud C., Yamanaka Y., Pawson T., Rossant J. (2006). Early lineage segregation between epiblast and primitive endoderm in mouse blastocysts through the Grb2–MAPK pathway. Dev Cell.

[34] Kurimoto K., Yabuta Y., Ohinata Y., Ono Y., Uno K.D., Yamada R.G. (2006). An improved single-cell cDNA amplification method for efficient high-density oligonucleotide microarray analysis. Nucleic Acids Res.

[35] Plusa B., Piliszek A., Frankenberg S., Artus J., Hadjantonakis A.K. (2008). Distinct sequential cell behaviours direct primitive endoderm formation in the mouse blastocyst. Development.

[36] Artus J., Piliszek A., Hadjantonakis A.K. (2011). The primitive endoderm lineage of the mouse blastocyst: sequential transcription factor activation and regulation of differentiation by Sox17. Dev Biol.

[37] Chazaud C., Yamanaka Y. (2016). Lineage specification in the mouse preimplantation embryo. Development.

[38] Krupa M., Mazur E., Szczepanska K., Filimonow K., Maleszewski M., Suwinska A. (2014). Allocation of inner cells to epiblast *vs* primitive endoderm in the mouse embryo is biased but not determined by the round of asymmetric divisions (8→16- and 16→32-cells). Dev Biol.

[39] Saiz N., Grabarek J.B., Sabherwal N., Papalopulu N., Plusa B. (2013). Atypical protein kinase C couples cell sorting with primitive endoderm maturation in the mouse blastocyst. Development.

[40] Graham S.J., Wicher K.B., Jedrusik A., Guo G., Herath W., Robson P. (2014). BMP signalling regulates the pre-implantation development of extra-embryonic cell lineages in the mouse embryo. Nat Commun.

[41] Johnson M.H., McConnell J.M. (2004). Lineage allocation and cell polarity during mouse embryogenesis. Semin Cell Dev Biol.

[42] Pratt H.P., Ziomek C.A., Reeve W.J., Johnson M.H. (1982). Compaction of the mouse embryo: an analysis of its components. J Embryol Exp Morphol.

[43] Samarage C.R., White M.D., Alvarez Y.D., Fierro-Gonzalez J.C., Henon Y., Jesudason E.C. (2015). Cortical tension allocates the first inner cells of the mammalian embryo. Dev Cell.

[44] Hirate Y., Hirahara S., Inoue K., Suzuki A., Alarcon V.B., Akimoto K. (2013). Polarity-dependent distribution of angiomotin localizes Hippo signaling in preimplantation embryos. Curr Biol.

[45] Leung C.Y., Zernicka-Goetz M. (2013). Angiomotin prevents pluripotent lineage differentiation in mouse embryos via Hippo pathway-dependent and -independent mechanisms. Nat Commun.

[46] Nishioka N., Inoue K., Adachi K., Kiyonari H., Ota M., Ralston A. (2009). The Hippo signaling pathway components Lats and Yap pattern Tead4 activity to distinguish mouse trophectoderm from inner cell mass. Dev Cell.

[47] Knoblich J.A. (2010). Asymmetric cell division: recent developments and their implications for tumour biology. Nat Rev Mol Cell Biol.

[48] Coulombe P.A., Wong P. (2004). Cytoplasmic intermediate filaments revealed as dynamic and multipurpose scaffolds. Nat Cell Biol.

[49] Paulin D., Babinet C., Weber K., Osborn M. (1980). Antibodies as probes of cellular differentiation and cytoskeletal organization in the mouse blastocyst. Exp Cell Res.

[50] Lim H.Y.G., Alvarez Y.D., Gasnier M., Wang Y., Tetlak P., Bissiere S. (2020). Keratins are asymmetrically inherited fate determinants in the mammalian embryo. Nature.

[51] Gueth-Hallonet C., Antony C., Aghion J., Santa-Maria A., Lajoie-Mazenc I., Wright M. (1993). Gamma-tubulin is present in acentriolar MTOCs during early mouse development. J Cell Sci.

[52] Howe K., FitzHarris G. (2013). A non-canonical mode of microtubule organization operates throughout pre-implantation development in mouse. Cell Cycle.

[53] Pomp O., Lim H.Y.G., Skory R.M., Moverley A.A., Tetlak P., Bissiere S. (2022). A monoastral mitotic spindle determines lineage fate and position in the mouse embryo. Nat Cell Biol.

[54] Chi F., Sharpley M.S., Nagaraj R., Roy S.S., Banerjee U. (2020). Glycolysis-independent glucose metabolism distinguishes TE from ICM fate during mammalian embryogenesis. Dev Cell.

[55] Dietrich J.E., Hiiragi T. (2007). Stochastic patterning in the mouse pre-implantation embryo. Development.

[56] Torres-Padilla M.E., Parfitt D.E., Kouzarides T., Zernicka-Goetz M. (2007). Histone arginine methylation regulates pluripotency in the early mouse embryo. Nature.

[57] Xu R., Li C., Liu X., Gao S. (2021). Insights into epigenetic patterns in mammalian early embryos. Protein Cell.

[58] Morgan H.D., Santos F., Green K., Dean W., Reik W. (2005). Epigenetic reprogramming in mammals. Hum Mol Genet.

[59] Goolam M., Scialdone A., Graham S.J.L., Macaulay I.C., Jedrusik A., Hupalowska A. (2016). Heterogeneity in Oct4 and Sox2 targets biases cell fate in 4-cell mouse embryos. Cell.

[60] Cui W., Cheong A., Wang Y., Tsuchida Y., Liu Y., Tremblay K.D. (2020). MCRS1 is essential for epiblast development during early mouse embryogenesis. Reproduction.

[61] Parfitt D.E., Zernicka-Goetz M. (2010). Epigenetic modification affecting expression of cell polarity and cell fate genes to regulate lineage specification in the early mouse embryo. Mol Biol Cell.

[62] Gillich A., Bao S., Grabole N., Hayashi K., Trotter M.W., Pasque V. (2012). Epiblast stem cell-based system reveals reprogramming synergy of germline factors. Cell Stem Cell.

[63] Yamaji M., Ueda J., Hayashi K., Ohta H., Yabuta Y., Kurimoto K. (2013). PRDM14 ensures naive pluripotency through dual regulation of signaling and epigenetic pathways in mouse embryonic stem cells. Cell Stem Cell.

[64] Ma Z., Swigut T., Valouev A., Rada-Iglesias A., Wysocka J. (2011). Sequence-specific regulator Prdm14 safeguards mouse ESCs from entering extraembryonic endoderm fates. Nat Struct Mol Biol.

[65] Burton A., Muller J., Tu S., Padilla-Longoria P., Guccione E., Torres-Padilla M.E. (2013). Single-cell profiling of epigenetic modifiers identifies PRDM14 as an inducer of cell fate in the mammalian embryo. Cell Rep.

[66] Hager G.L., McNally J.G., Misteli T. (2009). Transcription dynamics. Mol Cell.

[67] Phair R.D., Scaffidi P., Elbi C., Vecerova J., Dey A., Ozato K. (2004). Global nature of dynamic protein–chromatin interactions *in vivo*: three-dimensional genome scanning and dynamic interaction networks of chromatin proteins. Mol Cell Biol.

[68] Plachta N., Bollenbach T., Pease S., Fraser S.E., Pantazis P. (2011). Oct4 kinetics predict cell lineage patterning in the early mammalian embryo. Nat Cell Biol.

[69] Biase F.H., Cao X., Zhong S. (2014). Cell fate inclination within 2-cell and 4-cell mouse embryos revealed by single-cell RNA sequencing. Genome Res.

[70] Shi J., Chen Q., Li X., Zheng X., Zhang Y., Qiao J. (2015). Dynamic transcriptional symmetry-breaking in pre-implantation mammalian embryo development revealed by single-cell RNA-seq. Development.

[71] Mallanna S.K., Ormsbee B.D., Iacovino M., Gilmore J.M., Cox J.L., Kyba M. (2010). Proteomic analysis of Sox2-associated proteins during early stages of mouse embryonic stem cell differentiation identifies Sox21 as a novel regulator of stem cell fate. Stem Cells.

[72] Kuzmichev A.N., Kim S.K., D’Alessio A.C., Chenoweth J.G., Wittko I.M., Campanati L. (2012). Sox2 acts through Sox21 to regulate transcription in pluripotent and differentiated cells. Curr Biol.

[73] Guo H., Zhu P., Wu X., Li X., Wen L., Tang F. (2013). Single-cell methylome landscapes of mouse embryonic stem cells and early embryos analyzed using reduced representation bisulfite sequencing. Genome Res.

[74] Smallwood S.A., Lee H.J., Angermueller C., Krueger F., Saadeh H., Peat J. (2014). Single-cell genome-wide bisulfite sequencing for assessing epigenetic heterogeneity. Nat Methods.

[75] Zhu P., Guo H., Ren Y., Hou Y., Dong J., Li R. (2018). Single-cell DNA methylome sequencing of human preimplantation embryos. Nat Genet.

[76] Sen M., Mooijman D., Chialastri A., Boisset J.C., Popovic M., Heindryckx B. (2021). Strand-specific single-cell methylomics reveals distinct modes of DNA demethylation dynamics during early mammalian development. Nat Commun.

[77] Cokus S.J., Feng S., Zhang X., Chen Z., Merriman B., Haudenschild C.D. (2008). Shotgun bisulphite sequencing of the *Arabidopsis* genome reveals DNA methylation patterning. Nature.

[78] Wang L., Zhang J., Duan J., Gao X., Zhu W., Lu X. (2014). Programming and inheritance of parental DNA methylomes in mammals. Cell.

[79] Zhu C., Gao Y., Guo H., Xia B., Song J., Wu X. (2017). Single-cell 5-formylcytosine landscapes of mammalian early embryos and ESCs at single-base resolution. Cell Stem Cell.

[80] Gao Y., Li L., Yuan P., Zhai F., Ren Y., Yan L. (2020). 5-formylcytosine landscapes of human preimplantation embryos at single-cell resolution. PLoS Biol.

[81] Lieberman-Aiden E., van Berkum N.L., Williams L., Imakaev M., Ragoczy T., Telling A. (2009). Comprehensive mapping of long-range interactions reveals folding principles of the human genome. Science.

[82] Ranisavljevic N., Borensztein M., Ancelin K. (2021). Understanding chromosome structure during early mouse development by a single-cell Hi-C analysis. Methods Mol Biol.

[83] Flyamer I.M., Gassler J., Imakaev M., Brandao H.B., Ulianov S.V., Abdennur N. (2017). Single-nucleus Hi-C reveals unique chromatin reorganization at oocyte-to-zygote transition. Nature.

[84] Collombet S., Ranisavljevic N., Nagano T., Varnai C., Shisode T., Leung W. (2020). Parental-to-embryo switch of chromosome organization in early embryogenesis. Nature.

[85] Yao H., Gao C.C., Zhang D., Xu J., Song G., Fan X. (2023). scm^6^A-seq reveals single-cell landscapes of the dynamic m^6^A during oocyte maturation and early embryonic development. Nat Commun.

[86] Brannan K.W., Chaim I.A., Marina R.J., Yee B.A., Kofman E.R., Lorenz D.A. (2021). Robust single-cell discovery of RNA targets of RNA-binding proteins and ribosomes. Nat Methods.

[87] VanInsberghe M., van den Berg J., Andersson-Rolf A., Clevers H., van Oudenaarden A. (2021). Single-cell ribo-seq reveals cell cycle-dependent translational pausing. Nature.

[88] Hu W., Zeng H., Shi Y., Zhou C., Huang J., Jia L. (2022). Single-cell transcriptome and translatome dual-omics reveals potential mechanisms of human oocyte maturation. Nat Commun.

[89] Yan Z., An J., Peng Y., Kong S., Liu Q., Yang M. (2021). DevOmics: an integrated multi-omics database of human and mouse early embryo. Brief Bioinform.

[90] Guo F., Li L., Li J., Wu X., Hu B., Zhu P. (2017). Single-cell multi-omics sequencing of mouse early embryos and embryonic stem cells. Cell Res.

[91] Li L., Guo F., Gao Y., Ren Y., Yuan P., Yan L. (2018). Single-cell multi-omics sequencing of human early embryos. Nat Cell Biol.

[92] Gu C., Liu S., Wu Q., Zhang L., Guo F. (2019). Integrative single-cell analysis of transcriptome, DNA methylome and chromatin accessibility in mouse oocytes. Cell Res.

[93] Zhao C., Biondic S., Vandal K., Bjorklund A.K., Hagemann-Jensen M., Sommer T.M. (2022). Single-cell multi-omics of human preimplantation embryos shows susceptibility to glucocorticoids. Genome Res.

[94] Wang Y., Yuan P., Yan Z., Yang M., Huo Y., Nie Y. (2021). Single-cell multiomics sequencing reveals the functional regulatory landscape of early embryos. Nat Commun.

[95] Yan R., Gu C., You D., Huang Z., Qian J., Yang Q. (2021). Decoding dynamic epigenetic landscapes in human oocytes using single-cell multi-omics sequencing. Cell Stem Cell.

[96] Tarkowski A.K. (1959). Experiments on the development of isolated blastomers of mouse eggs. Nature.

[97] Krawczyk K., Kosyl E., Czescik-Lysyszyn K., Wyszomirski T., Maleszewski M. (2021). Developmental capacity is unevenly distributed among single blastomeres of 2-cell and 4-cell stage mouse embryos. Sci Rep.

[98] Maemura M., Taketsuru H., Nakajima Y., Shao R., Kakihara A., Nogami J. (2021). Totipotency of mouse zygotes extends to single blastomeres of embryos at the four-cell stage. Sci Rep.

[99] Tang F., Barbacioru C., Wang Y., Nordman E., Lee C., Xu N. (2009). mRNA-seq whole-transcriptome analysis of a single cell. Nat Methods.

[100] Tang F., Barbacioru C., Nordman E., Bao S., Lee C., Wang X. (2011). Deterministic and stochastic allele specific gene expression in single mouse blastomeres. PLoS One.

[101] Xue Z., Huang K., Cai C., Cai L., Jiang C.Y., Feng Y. (2013). Genetic programs in human and mouse early embryos revealed by single-cell RNA sequencing. Nature.

[102] Yan L., Yang M., Guo H., Yang L., Wu J., Li R. (2013). Single-cell RNA-seq profiling of human preimplantation embryos and embryonic stem cells. Nat Struct Mol Biol.

[103] Deng Q., Ramskold D., Reinius B., Sandberg R. (2014). Single-cell RNA-seq reveals dynamic, random monoallelic gene expression in mammalian cells. Science.

[104] Blakeley P., Fogarty N.M., del Valle I., Wamaitha S.E., Hu T.X., Elder K. (2015). Defining the three cell lineages of the human blastocyst by single-cell RNA-seq. Development.

[105] Fan X., Zhang X., Wu X., Guo H., Hu Y., Tang F. (2015). Single-cell RNA-seq transcriptome analysis of linear and circular RNAs in mouse preimplantation embryos. Genome Biol.

[106] Mohammed H., Hernando-Herraez I., Savino A., Scialdone A., Macaulay I., Mulas C. (2017). Single-cell landscape of transcriptional heterogeneity and cell fate decisions during mouse early gastrulation. Cell Rep.

[107] Posfai E., Petropoulos S., de Barros F.R.O., Schell J.P., Jurisica I., Sandberg R. (2017). Position- and Hippo signaling-dependent plasticity during lineage segregation in the early mouse embryo. Elife.

[108] Zhang Y., Yan Z., Qin Q., Nisenblat V., Chang H.M., Yu Y. (2018). Transcriptome landscape of human folliculogenesis reveals oocyte and granulosa cell interactions. Mol Cell.

[109] Leng L., Sun J., Huang J., Gong F., Yang L., Zhang S. (2019). Single-cell transcriptome analysis of uniparental embryos reveals parent-of-origin effects on human preimplantation development. Cell Stem Cell.

[110] Singh M., Widmann T.J., Bansal V., Cortes J.L., Schumann G.G., Wunderlich S. (2019). The selection arena in early human blastocysts resolves the pluripotent inner cell mass. BioRxiv.

[111] Starostik M.R., Sosina O.A., McCoy R.C. (2020). Single-cell analysis of human embryos reveals diverse patterns of aneuploidy and mosaicism. Genome Res.

[112] Ren Y., Yan Z., Yang M., Keller L., Zhu X., Lian Y. (2022). Regional and developmental characteristics of human embryo mosaicism revealed by single cell sequencing. PLoS Genet.

[113] Fan X., Tang D., Liao Y., Li P., Zhang Y., Wang M. (2020). Single-cell RNA-seq analysis of mouse preimplantation embryos by third-generation sequencing. PLoS Biol.

